# The effect of COVID-19 on employees' mental health

**DOI:** 10.1038/s41598-022-18692-w

**Published:** 2022-09-05

**Authors:** Didem Rodoplu Şahin, Mustafa Aslan, Harun Demirkaya, Hülya Ateşoğlu

**Affiliations:** 1 Kocaeli University, İzmit, Türkiye; 2 Istanbul Gelisim University, Istanbul, Türkiye

**Keywords:** Psychology, Human behaviour

## Abstract

Long lockdowns, food shortages, and the inability to receive basic primary healthcare have aggravated the effects of pandemics. However, most studies have focused on the health problems of the infected people or the measures employed to keep the disease under control. This cross-sectional study focused primarily on the mental health issues of employees. By employing a convenient sampling method, we reached 237 respondents (135 with coronavirus history) to assess the impact of the pandemic on employees. Multivariate causal relationships were assessed with Structural Equation Modeling (SEM). The predictors included internal entrapment (INT) and difficulty identifying feelings (DIF), which are significant predictors of depression (DEPR). DIF was found to be a significant predictor of INT and EXT feelings, while FEAR was found to be a significant predictor of INT, DIF, and DEPR. Quality of life (QoL) was found to be a significant predictor of DIF and DDF, DEPR, EXT and INT, and FEAR. The results also showed that DIF mainly manifested its effect on depression through INT. The DEPR level of employees working only from home was higher than that of other employees. The depression levels of women, young employees, and those whose QoL was adversely affected by the coronavirus were higher than the rest.

## Introduction

The COVID-19 pandemic, which emerged in December 2019, caused a public health crisis worldwide. On July 28, 2022, the number of confirmed cases was over 571 million, and the death toll was over 6.3 million worldwide^1^; these figures continue to rise steadily every day. The situation was not different in Turkiye, with over 15 million confirmed cases and around one hundred thousand deaths due to the coronavirus between March 2020 and May 2022^2^. This situation has created a sense of threat and concern that has spread at an alarming rate over the world^3^. Due to COVID-19, millions of people were locked down for weeks, lost their jobs, and, most importantly, their loved ones. In short, COVID-19 has had an impact on all aspects of our lives, from financial to social interactions, family to business relations, and physical to mental health, especially healthcare workers who have been fighting coronavirus at the frontline^4,5^, and older adults^6^.

Many COVID-19 patients show signs of post-traumatic stress disorder (PTSD). Employees suffering from PTSD are more likely to suffer from other mental health concerns and lose their ability to focus on cognitive activities. The three main clusters of PTSD symptoms (avoidance, intrusion, and hyperarousal) were proved to be substantially predicted by high levels of alexithymia, dissociation, anxiety, and sadness in persons who had recovered from COVID-19^7^. People who suffer from PTSD harbor frequent intense, distressing thoughts and feelings related to their traumatic experience. They may be stricken with grief, fear, or rage or feel isolated or disconnected from others^8^. They are also more likely to have alexithymic characteristics^9^, which means they can think, act, communicate, and perceive emotions but fail to correlate them with the related feelings, i.e., disconnection of the body and mind. In the absence of communication between the mind and the body, a state of body-mind dissociation ensues, which might be a sign of a protective mechanism or an alexithymic disorder^10^.

Moreover, the pandemic increased the risk of PTSD. According to Total Brain and the National Alliance of Healthcare Purchaser Coalitions^11^**,** the risk of PTSD has climbed month over month and, as of June 2022, is 51% greater than in the pre-pandemic era. According to the measure, nearly one-fifth of employees were at risk of having PTSD.

*Alexithymia* is a Greek word that means "*absence of words for emotions*"^12^. People suffering from this disorder have problems establishing a correlation between their feelings and thoughts and expressing them^12^. People with alexithymia typically struggle to recognize and express their emotions, display emotional functions, and establish interpersonal relationships^13^. Alexithymia has three dimensions: (1) difficulty in identifying feelings and distinguishing them from bodily sensations (DIF); (2) difficulty in describing feelings and putting them into words (DDF); (3) externally oriented thinking (EOT)^14^. The dimensions associated with identifying (DIF) and describing (DDF) feelings are positively related to depression and anxiety^15–18^.

Entrapment, on the other hand, is the desire to leave an unpleasant or challenging circumstance or uncertainty while also feeling compelled to avoid the unpleasantness of unease. When an individual is subjected to prolonged stress or has his or her conduct restricted by internal or external circumstances, he or she is said to be entrapped. Feeling entrapped in all aspects of life can have a negative impact on one's self-development and interpersonal relationships, either directly or indirectly^19^. Furthermore, when a person feels impotent to modify his or her circumstances, he or she is more likely to develop mental health issues. The experience of entrapment can be caused by a variety of factors, which the research categorizes as internal and external aspects^20,21^. During COVID- 19, people all around the world have had more than their fair share of suffering from feelings of entrapment^22^.

During the COVID-19 pandemic, many people died, and many went through traumatic experiences. Despite the fact that the number of confirmed cases is limited to barely 3.5% of the world's population, the coronavirus outbreak has caused increased fear and trauma due to widespread media coverage. Prolonged lockdowns, both qualitative and quantitative job insecurities and unemployment due to downsizing or bankruptcies^22^, shortage of food, inability to receive basic healthcare, fear of death, or causing the death of a loved one were primary factors are worsening the trauma. The outbreak, which rendered many people unable to think, act, or react^24,25^, has become this generation's worst frustrating experience. People were unable to avoid this predicament despite their best efforts^20,21^. They felt helpless^26,27^ and defeated^28^, no matter the measures were taken against the disease.

Although symptoms and the effects of coronavirus on health have been excessively covered by media^29^, many people, including healthcare workers, experienced vaccine hesitation due to a lack of confidence in the vaccines^30,31^. Moreover, misinformation spread through social media^31^, and being exposed to them due to the overuse of electronic devices during the lockdowns left people anxious, fearful, and ultimately hopeless^32^. Furthermore, the lockdown and other measures to keep the disease under control restricted people's engagement in physical and social activities, making them vulnerable to a higher risk of physical and physiological problems^33^.

Despite the pandemic's highly unfavorable impact on public health, most of the research on the COVID-19 pandemic has been mainly on infected people's health issues or the impact of lockdowns or other measures used to keep the disease under control. Although every adult is either an employee or has at least one employee in their household, there has been almost no research comparing the mental health of infected and uninfected employees and examining the impact of the pandemic as a whole (not only the steps taken to prevent the spread of coronavirus but also the fear experienced by individuals).

Hence, this study aims to investigate the effect of COVID-19 on employees' mental health by assessing the effect of coronavirus-caused fear on *identifying* and *describing feelings* dimensions of *alexithymia* and of *entrapment* on *depression level* of employees. Although workers of some sectors, especially the health care sector, are exposed to COVID-19 more than any other sector, this study does not focus on a specific sector, gender, or age group.

## Methods

### Study design

The questionary consists of five parts. The questions in the first part were about the demographics of the participants, such as age, gender, and workplace during the pandemic (e.g., from home, both from home & workplace, and only from the workplace).

The second part incorporated a questionnaire including the following questions: "Have you lost any relatives or close friends due to coronavirus?", "How did the COVID-19 pandemic affect the quality of your life?", and "What would you fear most if you had been infected with coronavirus?" In the third part, we used three different scales: (1) the Turkish version of the Toronto Alexithymia Scale (TAS-20)^34^ to measure *identifying* and *describing feelings*, (2) the 21-item Beck Depression Inventory-II^35^, and (3) the entrapment scale developed by Gilbert and Allan^36^. The scores of the Beck Depression Inventory-II were interpreted as suggested by Smarr and Keefer^37^.

#### Fear

Because the current scales have not been measuring the fear of infecting and causing the death of other people, especially relatives and loved ones, the authors have to create their own scale. The scale had a total of five items, and the respondents were asked to rate the five items from 1 to 5 (From the lowest to the highest, the level of fear is ranked from 1 to 5). The exact same questions were asked to both groups but with different tenses (e.g., "I am afraid of dying" to uninfected, and "I was afraid of dying" to the employee with coronavirus history).

The five items were related with:

- Afraid of being infected with the coronavirus.
- Afraid of infecting one's own family members or loved ones.
- Afraid of infecting people other than one's own family members and loved ones.
- Afraid of losing someone because of transmitting the disease to him or her.
- Afraid of dying.

The aim of asking the question, "How did the COVID-19 pandemic affect the quality of your life?" was to assess the participant's perception of the quality of life (QoL) during the pandemic. The options given to the question were (1) no effect; (2) minimal adverse effect; (3) moderate adverse effect; (4) very high adverse effect.

Participants marked (0) if they had not lost any relatives or close friends, (1) for one relative or close friends, and (2) for more than one relative and close friends.

### Data collection

This cross-sectional study was conducted in the Istanbul province of the Republic of Turkiye between September 26 and October 15, 2021. We reached the participants through social media and sent the questionary links (a total of two links; one for those with coronavirus history and one for those having no history) to those who volunteered to participate in the study. To promote participation, the researchers undertook to donate to Darülşşafaka Society-a well-known NGO in Turkiye founded in 1863 to provide equality of opportunity in education to needy, talented children who had lost their fathers—and stated this undertaking in the introduction part of the questionary. Participants then shared the questionary in their networks.

Being an employee (18 years old or over) was required to take part in the study. 237 of the 243 collected surveys were included in the analysis. Six surveys were omitted because two of the participants were underage, and four were housewives and thus were not eligible to participate in the study. The participants' average age was 40.17. Table 1 shows the demographics of the participants.

**Table 1:** Demographics of the participants.

	Options	Coding	No COVID-19 History	With COVID-19 History	Total	%
COVID History	No	0	102	–	102	43.00
	Yes	1	–	135	135	57.00
Age	Below 20	1	2	2	4	1.70
	21–25	2	9	9	18	7.60
	26–30	3	12	25	37	15.60
	31–35	4	7	21	28	11.80
	36–40	5	17	22	39	16.50
	41–45	6	14	12	26	11.00
	46–50	7	17	22	39	16.50
	51–55	8	11	14	25	10.50
	56 and Above	9	13	8	21	8.90
Gender	Male	1	45	56	101	42.60
	Female	2	57	79	136	57.40
Decease of a Relative or Acquaintance	None	0	51	74	125	52.70
	One	1	22	31	53	22.40
	Two or More	2	29	30	59	24.90
Quality of Life	No Effect	0	91	33	124	52.30
	Minimal Adverse Effect	1	0	35	35	14.80
	Moderate Adverse Effect	2	5	46	51	21.50
	Very High Adverse Effect	3	6	21	27	11.40
Work Location	Home Only	1	25	20	45	19.00
	Both Home & Workplace	2	40	48	88	37.10
	Workplace Only	3	37	67	104	43.90
	Total		102	135	237	100.0

This study is approved by the Kocaeli University Social and Human Sciences Ethics Committee (protocol number: E-10017888–108.99–62,960).

### Data analysis

**Figure 1:**
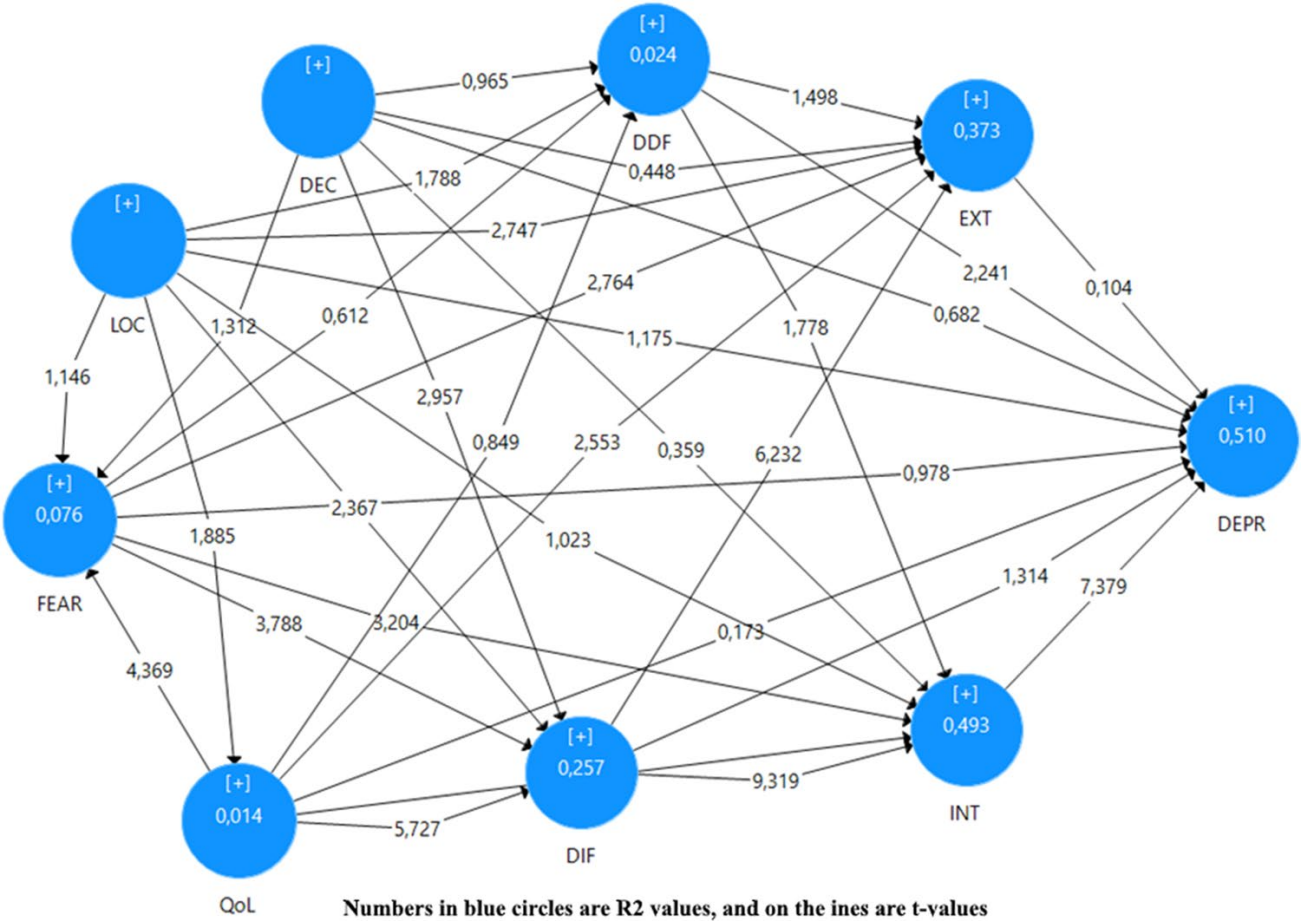
Path model. FEAR: Fear; LOC: Work Location; DEC: Decease of a Relative or Acquaintance; QoL: Quality of Life; DDF: Difficulty Describing Feelings; DIF: Difficulty Identifying Feelings; EXT: External Entrapment; INT: Internal Entrapment; DEPR: Depression Level.

Confirmatory Composite Analysis, Convergence and Discriminant Validities, and Reliability Tests were performed. Partial Least Square Structures Equation Modelling (PLS-SEM) with SmartPLS version 3.2.9 is used for data analysis. The coefficient of determination (R-square) and the Q-square value (the prediction relevance) was used to assess the model's acceptability. SPSS version 26 was also used as deemed necessary. The Pearson correlation coefficient and significance levels used to interpret results of correlation analysis. Figure 1 depicts path analyses using the path model.

Criteria for the validity and reliability are as follow:

- For Convergence Validity:
  - The average Variance Extracted (AVE) value must be equal to or greater than 0.50^38,39^.
  - Composite Reliability (CR) value must be equal to or greater than 0.70 and the square root of the AVE value^38,39^.
  - Cronbach Alpha value must be equal to or greater than 0.70^38,39^.
- For Discriminant Validity:
  - Heterotrait-Monotrait Ratio (HTMT) Values have to be 0.90 for the theoretical concepts close to each other and 0.85 for those that are distinct^40^.
  - Variance Inflation Factor (VIF) value must be below 5^41^.
- For model acceptability:
  - The coefficients of determination (R^2^), which implies the model's goodness-of-fit for the dependent variable, must be greater than 0.10^39,42^.
  - The Q^2^ value (the prediction relevance) has to be greater than zero^43^.

Furthermore, factor loadings have to be equal to or greater than 0.70. and the items with factor loadings below 0.40 have to be excluded from the analysis. Items with factor loadings between 0.40 and 0.70 will be kept in the model if CR and Cronbach Alpha values of the construct are over the threshold^39^.

### Background of the study

A close friend of one of the article's authors called in and asked for assistance about quitting his job. Following a discussion on how he felt, he appeared to be perplexed and had difficulty articulating and expressing his emotions. Afterwards, the author interviewed approximately 20 persons with coronavirus history, and observed similar symptoms with majority of them. Especially those experienced the coronavirus severely had difficulty with describing and expressing their feelings. Most of them described the situation they are in as "I feel like I'm being suffocated." Based on their observations, the authors designed this study to investigate the possible effect of coronavirus on alexithymia, entrapment, and depression.

### Ethical approval

This study is approved by the Kocaeli University Social and Human Sciences Ethics Committee (protocol number: E-10017888-108.99-62,960).

### Human and animal rights

All procedures performed in studies involving human participants were in accordance with the ethical standards of the institutional and/or national research committee and with the 1964 Helsinki declaration and its later amendments or comparable ethical standards.

### Informed consent

Informed consents were obtained from all individuals participated in the study.

## Results and discussion

After performing Confirmatory Composite Analysis (CCA), the factor loading of item number 11 of the TAS-20 was found to be less than 0.40 and removed from the model. The final run's Cronbach's Alpha, CR, and AVE values (Table 2) confirmed that the scales collectively satisfied the internal consistency reliability and convergent validity conditions.

**Table 2:** Cronbach's Alpha. CR. and AVE Values.

Construct	Cronbach's Alpha	CR	AVE
DDF (Difficulty Describing Feelings)	0.807	0.871	0.693
DIF (Difficulty Identifying Feelings)	0.908	0.927	0.620
EXT (Feelings of External Entrepment)	0.955	0.962	0.717
FEAR	0.873	0.903	0.654
INT (Feeling of Internal Entrapment	0.969	0.972	0.702
DEPR (Depression)	1.000	1.000	1.000
DEC (Decease of )	1.000	1.000	1.000
LOC (Work Location)	1.000	1.000	1.000
QoL (Quality of Life)	1.000	1.000	1.000

The highest HTMT value was measured as 0.751 < 0.900 between INT and EXT, and VIF as 2.817 < 5.000 between INT and DEPR. Therefore, we concluded that the scales satisfied the discriminant validity condition, and no collinearity was observed between variables.

The coefficients of determination (R^2^), which imply the model's goodness-of-fit, and Q^2^ value (the prediction relevance), were measured and reported in Table 3.

**Table 3:** R^2^ and Q^2^ Values of Measurement Model.

	R Square	Q Square
DDF	0.024	0.009
DEPR	0.510	0.460
DIF	0.258	0.154
EXT	0.373	0.257
FEAR	0.076	0.039
INT	0.494	0.338

The results reported in Table 3 show that the Q^2^ and R^2^ values meet the criteria. Hence, it was concluded that the measurement model was acceptable.

Following the verification of the validity, reliability of the scales, and the measurement model's acceptance, The Partial Least Squares Structural Equation Modeling (PLS-SEM) and path analysis were used to test the structural equation model. In the analyses, using the bootstrapping method, 5,000 sub-samples were taken. The path coefficients (β values) and statistical significance (*p* values) of the effects were calculated and reported in Table 4 (only significant effects included in the table, full table provided as supplementary resource). The total effects were also calculated and reported in Table 5.

**Table 4:** Direct and Indirect Effects (*p < 0.05; **p < 0.01).

Path	β
QoL → FEAR → INT → DEPR	0.020*
QoL → FEAR → INT	0.033**
QoL → FEAR → EXT	0.036*
QoL → FEAR → DIF → INT → DEPR	0.018*
QoL → FEAR → DIF → INT	0.030**
QoL → FEAR → DIF → EXT	0.022*
QoL → FEAR → DIF	0.051**
**QoL** → **FEAR**	0.250**
**QoL** → **EXT**	0.163*
**QoL** → **DIF** → **INT** → **DEPR**	0.125**
**QoL** → **DIF** → **INT**	0.204**
**QoL** → **DIF** → **EXT**	0.150**
**QoL** → **DIF**	0.350**
**LOC** → **EXT**	0.138**
LOC → DIF → INT → DEPR	− 0.049*
LOC → DIF → INT	− 0.079*
LOC → DIF → EXT	− 0.058*
**LOC** → **DIF**	− 0.137*
DEC → DIF → INT → DEPR	0.060*
DEC → DIF → INT	0.098**
DEC → DIF → EXT	0.072**
**DEC** → **DIF**	0.168**
FEAR → INT → DEPR	0.081**
**FEAR** → **INT**	0.132**
**FEAR** → **EXT**	0.142**
FEAR → DIF → INT → DEPR	0.073**
**FEAR** → **DIF** → **INT**	0.118**
FEAR → DIF → EXT	0.087**
**FEAR** → **DIF**	0.203**
**INT** → **DEPR**	0.614**
**DIF** → **DEPR**	0.140*
**DIF** → **INT**	0.582**
**DIF** → **INT** → **DEPR**	0.358**
**DIF** → **EXT**	0.428**
**DDF** → **DEPR**	− 0.108*

Significant values are in bold.

**Table 5:** Total effects.

	DDF	DEC	DEPR	DIF	EXT	FEAR	INT	LOC	QoL
DDF			− **0.160**		− 0.088		− 0.084		
DEC	− 0.073		0.055	**0.185**	0.074	0.084	**0.108**		
DEPR									
DIF			**0.455**		**0.427**		**0.582**		
EXT			0.008						
FEAR	− 0.046		**0.138**	**0.204**	0.233		**0.255**		
INT			**0.614**						
LOC	**0.115**		− 0.087	− **0.105**	0.096	− 0.046	− 0.018		**0.117**
QoL	− 0.067		**0.272**	**0.401**	**0.376**	**0.250**	**0.366**		

Significant values are in bold.

As per the results reported in Tables 4 and 5, Depression (DEPR) was increased mainly by Internal Entrapment (INT), Difficulty Identifying Feelings (DIF), and Quality of Life (QoL), but Difficulty Describing Feelings (DDF) reduced it. INT was mainly affected by DIF, QoL, and FEAR. FEAR was mainly affected by QoL, DIF by QoL, FEAR, and Decease of a Relative or Acquaintance (DEC), respectively, and External Entrapment (EXT) by DIF. Moreover, Quality of Life (QoL) was affected by work location (LOC).

When an individual's perceived quality of life is being affected adversely, it causes an increase in the difficulty of identifying feelings, fear, external and internal entrapment feelings, and ultimately depression. The perceived level of quality of life is affected by work location. The perceived quality of life of the individual working from home is better than those working only from a workplace.

The work location also affects the external entrapment (Table 4), which makes us think that the measures taken to prevent the spread of the pandemic cause employees working only from the workplace to feel trapped in a situation they cannot escape. Interestingly enough, the work location did not have a significant effect on employees' fear. Upon not being able to observe any effect of work location on fear, we took a step forward and performed a correlation analysis. Still, we could not determine any correlation (r = − 0.043; *p* = 0.516). Normally working at the workplace, which requires being among crowds (especially during the rush hour commutes) every day, should have caused employees to develop a certain level of fear of contracting coronavirus and transmitting it to other people, whether they be family members or others. However, the results of our study showed the other way around, i.e., the fear of coronavirus has no relation to the workplace.

The average fear is measured as 4.0153 out of 5 point scale. As per descriptive statistics given in Table 6, the highest fear employees experienced was causing someone else's death because of transmitting the disease to him or her (M = 4.4153), and the lowest was dying (M = 2.7089).

**Table 6:** Fear.

Item	COVID History	N	M	SD	N	Min	Max	M	SD
Afraid of dying	No	102	2.8137	1.51383	237	1.00	5.00	2.7089	1.5306
	Yes	135	2.6296	1.54408					
Afraid of infecting people other than his/her own family members and loved ones	No	102	4.1961	1.22708	237	1.00	5.00	4.2785	1.1192
	Yes	135	4.3481	1.17383					
Afraid of being infected with the coronavirus	No	102	4.2745	1.13589	237	1.00	5.00	4.2827	1.1969
	Yes	135	4.4963	1.02850					
Afraid of infecting his/her family members or loved ones	No	102	4.1078	1.17656	237	1.00	5.00	4.4008	1.0793
	Yes	135	4.4074	1.06011					
Afraid of causing someone else's death because of transmitting the disease to him or her	No	102	4.1275	1.25605	236	1.00	5.00	4.4153	1.0863
	Yes	134	4.6343	0.88037					
**FEAR (Aggregated)**	**No**	**102**	**3.9039**	**1.06417**	**236**	**1.00**	**5.00**	**4.0153**	**0.96055**
	**Yes**	**134**	**4.1000**	**0.86806**					

Significant values are in bold.

The mean differences of the following items were statistically significant compared to those with coronavirus history:

- Afraid of infecting his/her family members or loved ones (ΔM = 0.30; p = 0.044)
- Afraid of causing the death of someone else because of transmitting the disease to him or her (ΔM = 0.51; p = 0.001).

In both items, the average was higher for employees with coronavirus history. Nevertheless, the mean difference of the overall fear was not statistically significant (p = 0.112). The mean differences among work-location groups were not statistically significant either.

Although the work location did not have a statistically significant effect on fear, it had a significant effect on difficulty identifying feelings (β = 0.138; p < 0.01) and on external entrapment feeling (β = -0.137; p < 0.05). Working only from home increases the feeling of external entrapment, which should be quite a normal feeling since working from home may cause employees to develop a sense of isolation and entrapment^44^. The expectation, however, was quite the opposite since during the pandemic, home is generally considered a safe haven, while going out may be perceived as a threat.

Working only from home increases difficulty in identifying feelings. As can be found in the literature, working from home causes employees to develop negative feelings and agoraphobics^44^. The problem with the female employees working only from home reminds the problem described by Freidan[^45^:11] in her book, *The Feminine Mystique*:

“American women have luxuries that women in other times and lands never dreamed of; part of the strange newness of the problem is that it cannot be understood in terms of the age-old material problems of man: poverty, sickness, hunger, cold. The women who suffer this problem have a hunger that food cannot fill.”

Female employees working only from home had everything they needed, as American women had, but the problem was feeling trapped in the house. For male workers, it is not exactly the same but similar; as Ahrentzen[^44^:282] quotes from a homeworker, "*It's difficult to detach from things at home. I must get physically away. [Man, Adults Only].*"

Another factor that increases the difficulty of identifying feelings is the loss of someone. Alexithymia is linked to a defensive mechanism that seeks to limit difficult, intense, and negative emotions and avoid terrifying or intolerable feelings^46^. When an employee loses his/her relative or close friend, this defense mechanism may enter the equation to protect the individual from the loss's painful, negative, or powerful feelings. In other words, the death of a loved one may trigger the defense mechanism, which may cause the person to have difficulty identifying feelings.

This study also showed that fear causes difficulty identifying and describing feelings. The isolation and feeling of loneliness experienced in the midst of the pandemic may lead us to recall the basic anxiety, for the conditions we experience during the pandemic resemble, if not identical to, those experienced during the basic anxiety. The *basic anxiety* is defined by Horney[^47^:41] as "*the feeling a child has of being isolated and helpless in a potentially hostile world.*" This basic anxiety may also increase the use of this defense mechanism and fear. Fear, in return, may cause increased difficulty identifying feelings. Furthermore, on top of this pandemic situation that makes people feel entrapped^20^, the fear of infecting their families and others may cause the employees to develop anxiety, worry, helplessness, and uncertainty^48^. This fear may cause an increase in internal and external entrapment feelings, which is another finding of this study.

We also found that difficulty identifying and describing feelings, two dimensions of alexithymia that are closely associated with depression and anxiety^15–18^, also affect internal and external entrapment feelings. Furthermore, we believe this study also contributes to understanding the effect of alexithymia on depression. One possible mechanism is that difficulty identifying feelings increases internal entrapment, which in return causes an increase in depression. Although the literature is full of studies showing the relation of alexithymia with anxiety and depression, no study that links it to the feeling of entrapment exists. Therefore, the explanation that we have come up with maybe erroneous or have inadequacies. Being unable to identify feelings may cause employees to feel trapped inside because they could be capable of finding a solution or a way out if they were able to identify what they feel. That is why the difficulty identifying feelings has the highest effect (β = 0.582; p < 0.01) on inner entrapment feelings. This feeling of internal entrapment causes depression since it was found to be a significant predictor of depression (β = 0.614; p < 0.01). In their study carried out on 145 undergraduate students, Motan and Gençöz^49^ came up with similar findings, concluding that internal entrapment is a significant predictor of depression and anxiety. The reducing effect of difficulty describing feelings on depression, on the other hand, is a subject that needs to be clarified.

As per the results given in Table 7, no statistically significant differences were found between employees with and without coronavirus history. Furthermore, the mean depression score of employees with coronavirus history (M = 10.2444) is lower than the others (M = 11.5392), while this difference is not statistically significant (p = 0.326).

**Table 7:** Group statistics and ındependent sample test results.

Variable	COVID history	N	M	SD	df	t	p
INT	No	102	11.5392	10.55272	235	1.045	0.297
	Yes	135	10.2444	9.28365			
EXT	No	102	2.4951	1.00754	235	− 0.827	0.409
	Yes	135	2.6111	1.09247			
DIF	No	102	3.8676	.83006	235	− 0.837	0.404
	Yes	135	3.8500	.80007			
DDF	No	102	2.6229	1.13174	235	0.165	0.869
	Yes	135	2.4627	1.19537			
FEAR	No	102	2.6069	1.10984	234	− 1.558	0.121
	Yes	135	2.7415	1.33010			
DEPR	No	102	11.5392	10.55272	235	0.984	0.928
	Yes	135	10.2444	9.28365			

The mean depression score of employees working only from home (M = 14.0000) is higher than those working from both home and workplace (M = 9.3409) and only from the workplace (M = 10.6538) and this difference is statistically significant (F(2,234) = 3.421; *p* = 0.034). The depression score of employees working only from home shows that those employees suffer from mild depression symptoms.

The mean depression score of employees below the age of 21 (M = 26.5000) statistically (F(8,228) = 3.388; *p* = 0.01) differs from employees between the age of 31–35 (M = 8.9643), 41–45 (M = 7.8077), 46–50 (M = 8.8974), and 56-and above (M = 6.6190). Furthermore, personnel under the age of 21 exhibit moderate depression (M = 26.5000) symptoms, while those between the ages of 21 and 25 exhibit mild depression (M = 14.7778) symptoms.

The mean depression score of females (M = 12.6250) is higher than that of males (M = 8.3465), and this difference is statistically significant (p = 0.01). The mean depression score differences of employees whose quality of life was affected at moderate or very high levels were statistically significant (F(3,233 = 7.529; *p* = 0.000). Moreover, the depression score of employees whose quality of life was affected at a very high level (M = 17.0000) reveals that those employees are showing mild depression symptoms. The depression score of employees whose quality of life was affected at a moderate level (M = 13.2353) is at the edge of mild depression.

### Multi-group analysis (MGA)

MGA was also performed to determine if the path model has statistically significant differences across demographic groups. The multi-group analysis allows researchers to reveal any significant differences in group-specific parameter estimates between pre-defined data groups (e.g., outer weights, outer loadings, and path coefficients). As suggested by Henseler et al.^50^, group comparisons using structural equation modeling (SEM) without establishing the invariance of composite models can be misleading^50^. Therefore, before conducting MGA, Measurement Invariance of Composite Models (MICOM) was assessed with its procedure in three distinct steps. The MGA may be performed on variables that have two groups. In our study, it may be performed only for gender and COVID-History variables.

The MICOM process was performed with SmartPLS 3.2 statistical software with 5,000 permutations, a two-tailed test type at 0.05 significance level, 1,000 maximum iterations, and a 10^–7^ stop criterion. When running MICOM in SmartPLS, Step 1 of the procedure suggested by Hanseler et al.^50^ is automatically confirmed^52–54^. Step 2 and Step 3 of MICOM were performed by a permutation test.

As per the results, full measurement invariance was found to exist in overall composites for both variables. The MGA is performed for both variables, and its results are reported in Table 8.

**Table 8:** MGA results of the model.

Groups	Path	Δβ_1–2_
COVID HIST (1: uninfected; 2: with COVID-19 history)	QoL → DEPR	− 0.3803**
	FEAR → INT	0.2539**
	QoL → EXT	− 0.2674*
	QoL → DIF	− 0.2726*
	FEAR → EXT	0.2696*
	DDF → DEPR	− 0.2002*
GENDER (1: Male; 2: Female)	LOC → QoL	− 0.4009**

According to Table 8, the effect of the quality of life on depression, external entrapment, and difficulty identifying feelings was higher for employees with COVID-19 history. Fear had a more significant impact on internal and external entrapments in those not infected with the coronavirus. On the other hand, the effect of difficulty describing feelings on depression was higher for employees with a coronavirus history. These results suggest that the employees who have been infected with coronavirus feel less fear than those uninfected since they have experienced the coronavirus. This experience may have reduced the fear and entrapment caused by fear. However, the difficulty describing employees' feelings with coronavirus history was higher than in others. This finding is also supported by the findings of Ayaz and Dincer^55^.

### Limitations

The findings of this study should be interpreted in light of its limitations. Because all of the data in this study came from the same source, it is susceptible to common method variance error. The Harman single-factor test^56^ was performed to determine the magnitude of this error. The test result was 0.34973 (which is less than 0.50), which makes us conclude that the inaccuracy is acceptable. Another disadvantage of the study is that the interpretations and inferences may not be as precise or exact as intended because no comparable study has been detected in the literature.

This study also explored the history of infected and uninfected people, not the severity of their clinical course. Therefore, the results need to be interpreted accordingly.

Another limitation of this study has not differentiated participants based on their work area. Since not all workers were exposed to the same stress levels, the results may not be generalized for all sectors and should be interpreted cautiously.

This study is not longitudinal, but a cross-sectional study and the authors do not have pre-pandemic data of the participants. Therefore, the inferences related to alexithymia and its impact on the appearance of affective symptoms during the pandemic could result from previous personality traits or underlying mental disorders. Hence, the results should be interpreted accordingly.

Other drawbacks include using a convenience sampling strategy, relying only on self-reported measuring instruments, and carrying out the measurement only once. It may raise concerns about the findings' generalizability, selection bias, and causality inference difficulties.

Another drawback stems from the study's design. The data was acquired from the participants at a single point in time, and the researchers did not follow up with them over time. As a result, the findings should be carefully interpreted and validated through longitudinal research.

### Implications for Practice

The dramatic spread of COVID-19 has disrupted lives, communities, and businesses alike. In their efforts to adapt themselves to the new challenges posed by the pandemic and mitigate the COVID- 19 impact, businesses were forced to find ways to help their employees stay in the work process as much as possible. Designing the workflow for employees in a way to enable them to work from home was one of the approaches by which many businesses responded to the coronavirus crisis. However, as revealed by this study, this approach or solution harbors an unforeseen and unrecognized consequence; it may cause employees to develop mental health problems.

As the study suggests, young employees and women who work solely at home are the two groups that suffer from mental health problems more than others. Proper conditions created in a workplace deeply motivate and engage employees and impact their mood, drive, and mental health. On the other hand, the home lacks such favorable features facilitating professional working and thus brings its own set of challenges that can negatively impact the mood when used as a workplace. Furthermore, working from home may cause the line between work and private life to get blurred. Expectations of other family members while working at home may leave female employees frustrated or feelings of unease, particularly at times when they fail to respond with the required reflex. Interruption of work with a ring at the door (to receive cargo, essential daily needs of the home such as drinking water etc.), technical problems (internet connection, computer problems that need the support of an IT person), personal calls, dropping by visitors/relatives sometimes extending their visit overnight and other unanticipated home situations also put a strain on the minds of employees and contribute to the undesirable atmosphere home offers for working.

As with the case of young employees (especially those under 21), the possibility of living with parents, and thus most probably, being unable to live in a home with physical conditions addressing to personal preferences and comfort, may cause distress while working from home. The presence of younger siblings who disrupt the working atmosphere with their nuisance or wishes, non-availability of a proper working space not accommodating the comfort office furniture provides, etc., may make young employees one of the groups suffering from mental issues more than others. Meanwhile, some young employees may also feel frustrated from being unable to express their inconvenient conditions at home to their employers. Being overwhelmed with the fear of the pandemic and thus feeling the urge to work at home, they may continue to work at home and feel its psychological pressure against all odds.

When these home-related inconveniences are kept in mind, businesses need to address all these mostly unrecognized issues to provide a favorable working atmosphere for their employees working only from home. Supporting the employees, whether by financial means or others deemed necessary, to improve their physical conditions at home will relieve them and make them feel less depressed, ultimately making them experience fewer mental issues.

Meanwhile, if possible, offering hybrid working conditions (working both from home and the workplace) or reducing the workload (including work hours) of those working only from home should be considered and assessed.

As well to young employees and female employees, employees with coronavirus history also seem to have higher mental health problems. The mental issues of the employees in these categories should be addressed by special care or particular policies.

On the other hand, working with psychologists or encouraging employees to visit psychologists, especially during the COVID-19 pandemic, maybe another solution for organizations. Finally, for those who experience mental health problems during the pandemic, organizations should be more flexible with their employees and amend their policies and key performance indicators accordingly.

## Conclusion

The aim of this study was to investigate the effects of fear on identifying and describing feelings dimensions of alexithymia and dimensions of entrapment on the depression level of employees during the COVID-19 pandemic. The results show that internal entrapment and difficulty identifying feelings are the significant predictors of depression, whereas the difficulty identifying feelings is the significant predictor of internal and external entrapment feelings. Fear was the significant predictor of internal entrapment, difficulty identifying feelings, and depression. On the other hand, quality of life is a significant predictor of difficulties identifying and describing feelings, depression, external and internal entrapment, and fear.

The results also show that difficulty identifying feelings manifests its effect on depression mainly through internal entrapment.

This study also revealed that the depression level of the employees working only from home is higher than other employees. Moreover, the depression level of women, young employees, and those whose life quality was adversely affected by the coronavirus is higher than the rest.

## Data Availability

The datasets used and/or analysed during the current study available from the corresponding author on reasonable request.
